# Plant morphology, physiological characteristics, accumulation of secondary metabolites and antioxidant activities of *Prunella vulgaris* L. under UV solar exclusion

**DOI:** 10.1186/s40659-019-0225-8

**Published:** 2019-04-01

**Authors:** Yuhang Chen, Xuerong Zhang, Qiaosheng Guo, Liping Cao, Qin Qin, Chen Li, Miao Zhao, Wenming Wang

**Affiliations:** 1 College of Pharmaceutical Sciences, Chengdu Medical College, Chengdu, 610500 Sichuan China; 2 Institute of Chinese Medicinal Materials, Nanjing Agricultural University, Nanjing, 210095 Jiangsu, China; 3 Rice Research Institute, Sichuan Agricultural University, Chengdu, 611130 Sichuan China; 4 Shanghai Traditional Chinese Medicine Co., LTD., Shanghai, 200002 China

**Keywords:** *Prunella vulgaris* L., UV exclusion, Physiological traits, Secondary metabolites, Antioxidants

## Abstract

**Background:**

*Prunella vulgaris* L. has been an important medicinal plant for the treatment of thyroid gland malfunction and mastitis in China for over 2000 years. There is an urgent need to select effective wavelengths for greenhouse cultivation of *P. vulgaris* as light is a very important factor in *P. vulgaris* growth. Here, we described the effects of natural light (control) and UV solar exclusion on the morphological and physiological traits, secondary metabolites contents and antioxidant activities of *P. vulgaris*.

**Results:**

The results showed that UV solar exclusion resulted in remarkable alterations to morphological and biomass traits; significantly reduced the chlorophyll a, chlorophyll b and total chlorophyll contents; significantly enhanced the ratio of chlorophyll a to b; and significantly increased the carotenoid and anthocyanin contents in *P. vulgaris*. UV solar exclusion significantly increased the catalase (CAT) and peroxidase (POD) activities, increased superoxide dismutase (SOD) and ascorbate peroxidase (APX) activities and slightly decreased the glutathione (GSH) content. UV solar exclusion significantly increased the soluble sugar and H_2_O_2_ contents and increased the soluble protein content but significantly decreased the proline content and slightly decreased the MDA content. The secondary metabolite contents (total phenolics, rosmarinic acid, caffeic acid, hyperoside, ursolic acid and oleanolic acid) and in vitro antioxidative properties (DPPH· and ABTS·^+^scavenging activities) were significantly increased in *P. vulgaris* spicas under UV solar exclusion. Additionally, the total polysaccharide and total flavonoids contents were slightly increased by UV solar exclusion. The salviaflaside content was significantly reduced by UV solar exclusion.

**Conclusion:**

Our study demonstrated that *P. vulgaris* activates several antioxidant defence systems against oxidative damage caused by UV solar exclusion.

## Background

*Prunella vulgaris* L. (Labiatae) is an important medicinal plant that is typically found in Europe and Asia [1, 2]. Dried spicas of *P. vulgaris*, Prunellae Spica, are a standard medicinal material in the Chinese pharmacopoeia [3] and is occasionally used for the treatment of thyroid gland malfunction, mastitis, pulmonary tuberculosis, infectious hepatitis, and hypertension [4]. In recent years, demand for Prunellae Spica has increased steadily due to its medicinal importance. Approximately five million kilograms of Prunellae Spica is required every year for Chinese patent medicine [5]. In addition to its pharmaceutical industrial use, the dried spicas of *P. vulgaris* are manufactured as a functional herbal tea, the fresh leaves of *P. vulgari*s seedlings are consumed as a vegetable dish in southeast China and *P. vulgari*s plants are often used as ornamental plants for urban greenery in southern China [6–8].

Phenolic acids, flavonoids, triterpenes and polysaccharides are the most abundant types of active ingredients in *P. vulgaris* [9]. Rosmarinic acid and caffeic acid are the major phenolic components in Prunellae Spica [10, 11] and act as photo-protective agents to scavenge ROS produced by UV-B radiation [12–14]. Salviaflaside, formed by the intermolecular condensation of two phenylpropanoid derivative molecules, is an indicator component in Prunellae Spica [4]. Hyperoside is another major flavonoid component of *P. vulgaris* that mitigates oxidative damage due to ROS production [15]. Of the triterpenes, ursolic acid and oleanolic acid are the most prevalent in *P. vulgaris* and possess antioxidant, anti-allergic, anti-inflammatory and antitumour functions [16, 17]. In addition, *P. vulgaris* polysaccharides have been reported as potential antioxidant and immunomodulatory agents for complementary medicine [18].

Additionally, studies using specific filters for solar UV exclusion to remove UV radiation (280–400 nm) have generally focused on the impacts of UV-B on plant growth and morphology [19]. Recent studies in India and Brazil have reported that solar UV exclusion significantly affects the photosynthesis, growth, yield and quality of some crop and medicinal plants [20, 21]. These studies have revealed the importance of specific wavelengths for the cultivation of crop and medicinal plants.

Previous studies have described the influence of abiotic factors, such as heavy metals [22], drought [23], UV-B radiation [15], different soil nutrient concentrations [24, 25] and different spectral lights [26], on the productivity, physiological, and quality of *P. vulgaris*. However, no study in the literature has investigated the effects of UV solar exclusion on the growth and quality of *P. vulgaris*. Therefore, it is necessary to explore the effects of UV solar exclusion on the morphological traits, photosynthetic pigment contents, cellular defence system products, secondary metabolite biosynthesis, and antioxidant activities of ethanol extracts of *P. vulgaris.*

## Results

### Morphology and biomass

The morphological and biomass characteristics of *P. vulgaris* were studied under UV solar exclusion (Table 1). Branch number per plant, stem weight and whole-plant weight under UV solar exclusion were significantly increased compared to the results obtained with control treatment. Spica number per plant, spica length, root weight, leaf weight and spica weight under UV solar exclusion were increased with no significance compared to the results obtained with control treatment. Spica width was decreased under UV solar exclusion, but both differences were not significant.

**Table 1:** The effects of UV solar exclusion on the morphological and biomass traits of *P. vulgaris*

Characteristics	Control	UV solar exclusion
Branch number per plant	2.3 ± 2.1 b	5.2 ± 0.9 a
Spica number per plant	8.7 ± 3.3 ab	13.0 ± 6.0 a
Spica length (cm)	4.32 ± 1.01 a	4.86 ± 0.98 a
Spica width (cm)	1.21 ± 0.12 a	1.16 ± 0.11 a
Root weight (g plant^−1^)	0.32 ± 0.15 a	0.50 ± 0.21 a
Stem weight (g plant^−1^)	1.71 ± 0.40 b	2.84 ± 0.81 a
Leaf weight (g plant^−1^)	1.98 ± 0.79 ab	2.87 ± 1.40 a
Spica weight (g plant^−1^)	1.30 ± 0.50 ab	1.73 ± 0.73 a
Whole plant weight (g plant^−1^)	5.42 ± 1.37 b	8.10 ± 1.72 a

Each value is presented as the mean ± SD (n = 10). Different letters in lines indicate a significant difference at *p* < 0.05

### Photosynthetic pigment and anthocyanin contents

The photosynthetic pigment and anthocyanin contents were studied in *P. vulgaris* under UV solar exclusion (Table 2). The chlorophyll a, chlorophyll b and total chlorophyll contents were significantly reduced by UV solar exclusion. Meanwhile, the ratio of chlorophyll a and b and the carotenoid and anthocyanin contents were significantly enhanced by UV solar exclusion, but the ratio of chlorophyll a and b under UV solar exclusion was not significant.

**Table 2:** The effects of UV solar exclusion on the photosynthetic pigment and anthocyanin contents of *P. vulgaris* leaves

Pigment parameter	Control	UV solar exclusion
Chlorophyll a (mg g^−1^)	1.60 ± 0.12 a	1.29 ± 0.02 b
Chlorophyll b (mg g^−1^)	1.24 ± 0.24 a	0.84 ± 0.01 b
Total chlorophyll (mg g^−1^)	2.84 ± 0.36 a	2.13 ± 0.03 b
Chlorophyll a/b ratio	1.31 ± 0.17 ab	1.54 ± 0.01 a
Carotenoids (mg g^−1^)	0.06 ± 0.04 b	0.10 ± 0.00 a
Anthocyanin (A g^−1^ FW)	2.67 ± 0.11 b	2.88 ± 0.03 a

Each value is presented as the mean ± SD (n = 3). Different letters in lines indicate a significant difference at *p* < 0.05

### Antioxidant systems

The POD, CAT, SOD and APX activities and GSH content were studied in *P. vulgaris* under UV solar exclusion (Table 3). The POD, CAT, SOD and APX activities were increased under UV solar exclusion, and the difference between POD and CAT was significantly increased compared to the results obtained with control treatment. By contrast, the GSH content slightly decreased under UV solar exclusion.

**Table 3:** The effects of UV solar exclusion on the POD, CAT, SOD and APX activities and GSH content in *P. vulgaris* leaves

Antioxidant system	Control	UV solar exclusion
POD activity (U mg^−1^ proteins)	78.48 ± 3.31 b	112.73 ± 21.37 a
CAT activity (U mg^−1^ proteins)	38.09 ± 7.22 b	53.06 ± 7.28 a
SOD activity (U mg^−1^ proteins)	49.12 ± 9.36 a	53.91 ± 14.11 a
APX activity (U mg^−1^ proteins)	0.62 ± 0.10 a	0.80 ± 0.07 a
GSH content (mg g^−1^ proteins)	95.79 ± 4.21 a	90.39 ± 1.68 a

Each value is presented as the mean ± SD (n = 3). Different letters in lines indicate a significant difference at *p* < 0.05

### Soluble protein, soluble sugar, proline, H_2_O_2_, and MDA contents

The soluble protein, soluble sugar, proline, H_2_O_2_, and MDA contents were studied in *P. vulgaris* under UV solar exclusion and supplemental UV-B radiation (Table 4). The soluble sugar and H_2_O_2_ contents were significantly increased by UV solar exclusion. The soluble protein content was slightly increased by UV solar exclusion. The proline and MDA contents were reduced by UV solar exclusion, and the proline content was significantly reduced by UV solar exclusion.

**Table 4:** The effects of UV solar exclusion on the soluble protein, soluble sugar, proline, H_2_O_2_ and MDA contents in *P. vulgaris* leaves

Oxidative stress indicators	Control	UV solar exclusion
Soluble protein (g L^−1^ FW)	0.25 ± 0.01 a	0.27 ± 0.04 a
Soluble sugar (mg mg^−1^ proteins)	7.68 ± 0.30 b	9.26 ± 0.57 a
Proline (μg g^−1^ FW)	34.40 ± 1.20 a	29.42 ± 1.33 b
H_2_O_2_ (mmol g^−1^ proteins)	43.25 ± 1.66 b	61.14 ± 0.59 a
MDA (nmol mg^−1^ proteins)	44.01 ± 7.11 a	41.33 ± 5.07 a

Each value is presented as the mean ± SD (n = 3). Different letters in lines indicate a significant difference at *p* < 0.05

### Secondary metabolite contents

The secondary metabolite contents were studied in *P. vulgaris* under UV solar exclusion (Table 5). The total phenolic, total flavonoid, caffeic acid, rosmarinic acid and hyperoside contents were significantly increased under UV solar exclusion, but the increase in the total flavonoid content under UV solar exclusion was not significantly different compared to the results obtained with control treatment. The oleanolic acid and ursolic acid contents were significantly increased under UV solar exclusion, and the total polysaccharide content increased but was not significant. Additionally, the salviaflaside content was significantly reduced by UV solar exclusion.

**Table 5:** The effects of UV solar exclusion on the total phenolic, total flavonoid, rosmarinic acid, caffeic acid, hyperoside, total polysaccharide, salviaflaside, ursolic acid and oleanolic acid contents in *P. vulgaris* spicas

Secondary metabolites	Control	UV solar exclusion
Total phenolics (%)	1.30 ± 0.15 b	1.97 ± 0.28 a
Total flavonoids (%)	4.31 ± 0.52 a	4.96 ± 0.23 a
Rosmarinic acid (%)	0.28 ± 0.01 b	0.30 ± 0.00 a
Caffeic acid (%)	0.02 ± 0.00 b	0.02 ± 0.00 a
Hyperoside (%)	0.02 ± 0.00 b	0.04 ± 0.00 a
Total polysaccharides (%)	3.68 ± 0.47 a	4.09 ± 0.66 a
Salviaflaside (%)	0.12 ± 0.00 a	0.08 ± 0.00 b
Ursolic acid (%)	0.52 ± 0.02 b	0.62 ± 0.02 a
Oleanolic acid (%)	0.10 ± 0.01 b	0.13 ± 0.01 a

Each value is presented as the mean ± SD (n = 3). Different letters in lines indicate a significant difference at *p* < 0.05

### In vitro antioxidant activities

The in vitro antioxidant activities of ethanol extracts of *P. vulgaris* spicas were studied under UV solar exclusion (Table 6). The scavenging activities of DPPH· and ABTS·^+^ were significantly enhanced under UV solar exclusion compared to the results obtained with control samples. According to the scavenging DPPH· radical activity results, ethanol extracts of *P. vulgaris* spicas subjected to UV solar exclusion showed significantly higher free radical scavenging activities than control extracts. These results also indicate that the ethanol extracts of *P. vulgaris* spicas under UV solar exclusion demonstrated higher ABTS·^+^ free radical scavenging activities than control samples.

**Table 6:** The effects of UV solar exclusion and supplemental UV-B radiation on DPPH· and ABTS·^+^ radical scavenging activities in ethanol extracts of *P. vulgaris* spicas

Antioxidant activities	Control	UV solar exclusion
DPPH· (%)	63.66 ± 1.95 b	80.82 ± 0.22 a
TEAC (mmol L^−1^ trolox)	0.87 ± 0.03 b	0.96 ± 0.01 a

Each value is presented as the mean ± SD (n = 3). Different letters in lines indicate a significant difference at *p* < 0.05

## Discussion

In the present study, the effect of UV solar exclusion on the morphology, physiology, secondary metabolites and antioxidant activities of *P. vulgaris* were investigated. In our study, we observed significant increases in the branch number per plant, stem weight and whole-plant weight of *P. vulgaris* as well as increased spica numbers per plant, spica length, root weight, leaf weight and spica weight (except for a slight decrease in spica width) with UV solar exclusion, which is consistent with observations of other crop and medicinal plants [27]. Previous studies have shown that UV solar exclusion significantly increases PS II efficiency and the photosynthesis rate and induces remarkable increases in the carbonic anhydrase, ribulose-1,5-bisphosphate carboxylase (Rubisco) and nitrate reductase activities; this additional carbon and nitrogen fixation via solar UV exclusion facilitates improvements in both the biomass and economic yield of the plants [28]. Thus, *P. vulgaris* morphology and biomass productivity were increased by UV solar exclusion.

In this study, the decrease in the total chlorophyll levels with solar UV exclusion was attributable to the decreased synthesis of chlorophyll a and chlorophyll b. However, the extent of the reduction was greater for chlorophyll b than for chlorophyll a, causing an increase in the chlorophyll a/b ratio following exposure to UV solar exclusion. This result indicated that a higher Chla/Chlb ratio in *P. vulgaris* leaves facilitates adaptation to oxidative stress by maximizing the energy efficiency and allowing plants to maintain efficient and stable operation of photosynthesis under UV solar exclusion.

The present study showed that the carotenoid content was significantly enhanced under solar UV exclusion. Similarly, previous studies showed that the carotenoid levels in medicinal plants were increased under UV solar exclusion [29]. Anthocyanins are well-known antioxidants and ROS scavengers [30] and regulate photo-oxidative damages [31, 32]. The present study revealed that UV solar exclusion stimulated the production of anthocyanin in *P. vulgaris* leaves, which is consistent with the results of previous studies [33, 34]. Thus, the leaf cells of *P. vulgaris* that accumulate carotenoids and anthocyanins are much more effective at eliminating ROS induced by photo-oxidative stress than leaves exposed to control treatments.

In the enzymatic defence system, SOD accelerates the conversion of superoxide to hydrogen peroxide (H_2_O_2_), and POD and CAT catalyse H_2_O_2_ breakdown into H_2_O and O_2_ [35]. APX, an indispensable component of the ascorbate–glutathione cycle required to scavenge H_2_O_2_, is mainly produced in chloroplasts and other cell organelles to maintain the redox state of cells [36]. In addition, GSH is an important antioxidant that protects plant cells against oxidative damage and reacts directly with ROS [37, 38]. The activities of POD, SOD, CAT and APX were increased by UV solar exclusion. Similar results were obtained in a previous study [39]. We hypothesized that UV solar exclusion primarily enhances photo-oxidative damage to *P. vul*garis plants. By contrast, our results showed that UV solar exclusion caused a slight decrease in the GSH content. These results indicated that UV solar exclusion induced oxidative stress and caused a large amount of H_2_O_2_-induced damage to *P. vul*garis plant cells, rendering GSH less capable of responding to stress, which is consistent with the results of a previous study [40].

Interestingly, the total soluble protein content in the leaves of *P. vulgaris* increased in UV exclusion samples, which agrees with the results of an earlier study [41]. These results confirmed that UV light exclusion enhanced biomass accumulation and the protein content in leaves due to the increased area and number of leaves resulting from altered photomorphogenesis. The sugar content was enhanced to similar levels following exposure of *P. vulgaris* to UV solar exclusion. The efficient role of sugars as true ROS scavengers during abiotic stress has previously been demonstrated, and the synergistic interaction of sugars and phenolic compounds functions as an integrated redox system in plants to scavenge ROS and enhance stress tolerance [42, 43]. Additionally, these results indicate that soluble protein and soluble sugar are osmotic regulators, and plants adapt to oxidative stress through osmotic regulation by altering the co-accumulation of soluble protein and soluble sugar. Similar results were reported in previous study [44].

Reactive oxygen species accumulation may cause damage to biomolecules, leading to altered metabolic processes in plants. In the present study, the H_2_O_2_ content was significantly increased by UV solar exclusion than by control treatment, which implies photo-oxidative damage [45, 46]. Proline accumulation is one of the important non-enzymatic defence systems and this system protects plant cells against ROS generated by photo-oxidative stress in some medicinal plants. UV solar exclusion induce photoinhibition, which stimulates ROS generation as indicated by the presence of MDA-lipid peroxidation products. MDA is widely used as an indicator of physiological stress in plants and causes changes to cell membrane properties, such as enzyme activity, fluidity and ion transport [47]. UV solar exclusion induced photo-oxidative stress in *P. vulgaris*, but the increase in MDA production was lower, revealing the high tolerance of these plants to UV solar exclusion. Thus, although large amounts of H_2_O_2_ were induced by photo-oxidative stress, *P. vulgaris* plants did not show any significant physiological damage, suggesting the presence of strong repair mechanisms.

Phenolic compounds are a large category of secondary metabolites in *P. vulgaris* spicas [2], and the antioxidative activities of phenolic extracts from *P. vulgaris* plants scavenge ROS [48] and suppress lipoperoxidation in pharmacological models [49]. In this study, the total phenolic contents of *P. vulgaris* spicas under UV solar exclusion were significantly higher than those under control treatment. Similar results were previously obtained in other plants [50, 51]. *P. vulgaris* under UV exclusion treatment had a significantly higher H_2_O_2_ content than plants grown under the control treatment. ROS may be serve as mediators by initiating the biosynthesis of certain phenolic compounds [52]. These results suggest that increasing the total phenolic content of *P. vulgaris* effectively induces ROS scavenging and decreases the degree of membrane lipid peroxidation under UV solar exclusion.

Flavonoids are important secondary metabolites in *P. vulgaris* spicas [2], demonstrating significant antioxidant activities in in vivo experiments [53]. In the present study, the flavonoid contents in *P. vulgaris* spicas significantly increased under UV solar exclusion, suggesting that increased flavonoids in *P. vulgaris* protect the plants against H_2_O_2_ induced by UV solar exclusion. Similar findings were reported by previous studies [54–56].

Rosmarinic acid is a major phenolic component in *P. vulgaris* spicas [10], and the Chinese Pharmacopoeia considers the amount of rosmarinic acid to be a quality control marker for Spica Prunellae [3]. In our study, the rosmarinic acid content was significantly enhanced by UV solar exclusion in this study. Previous studies indicated that rosmarinic acid possesses excellent ROS-scavenging capability and is proposed to be a true photo-protective agent [57]. This photo-oxidative stress leads to the production of H_2_O_2_, which in turn activates and increases the levels of PAL (Phenylalanine-ammonia-lyase, a key enzyme in the biosynthesis of rosmarinic acid) [58], providing sufficient amounts of substrate to sustain higher levels of rosmarinic acid synthesis in metabolic pathways [59]. This result indicated that increased rosmarinic acid exerted a protective effect on *P. vulgaris* plants against ROS production induced by UV solar exclusion.

Caffeic acid is an important phenolic component in *P. vulgaris* spicas [15]. The 60% ethanol extract of *P. vulgaris* contains the major bioactive compounds containing caffeic acid and shows high antioxidant activity in in vitro and in vivo experiments [60]. High amounts of caffeic acid in *P. vulgaris* spica extracts indicate that the antioxidative activities of caffeic acid may be responsible for antinociceptive and antidiabetogenic effects in diabetic mice models [61]. In this study, the caffeic acid content in *P. vulgaris* spicas significantly increased under UV solar exclusion, which is consistent with previous reports [62]. These results suggest that the increased caffeic acid content observed in *P. vulgaris* spicas under photo-oxidative stress may prevent lipid peroxidation and scavenge ROS in *P. vulgaris* cells.

Hyperoside, the main flavonol glycoside component in *P. vulgaris* spicas [4], was found to inhibit H_2_O_2_-induced apoptosis in Chinese hamster lung fibroblast cells and increase the CAT and glutathione peroxidase (GSH-Px) activities [63]. In this study, the UV solar exclusion significantly promoted hyperoside accumulation in *P. vulgaris* spicas, which is consistent with the results of a previous study [64]. Previous studies also indicated that hyperoside was an effective ROS scavenger and demonstrated the highest DPPH· scavenging capability [65]. Therefore, increased hyperoside levels in *P. vulgaris* spicas may act as compensators to scavenge ROS generated from UV solar exclusion.

Polysaccharides are an important type of secondary metabolite in *P. vulgaris* spicas [2]. Recently, a study suggested that *P. vulgaris* polysaccharides could be explored as potential antioxidant and immunomodulatory agents for complementary medicine and may be candidates for the treatment of hypoimmunity and immunodeficiency diseases [18]. In this study, UV solar exclusion was beneficial for the accumulation of polysaccharides in *P. vulgaris*, especially when the plants were under the oxidative stress, which was similar to the results obtained in previous studies [66]. Polysaccharides effectively eliminate ROS induced by photo-oxidative stress, protecting DNA strand breaks and inhibiting membrane lipid peroxidation.

Salviaflaside is a major phenolic glycoside component in *P. vulgaris* spicas and possibly acts as a quality indicator for Spica Prunellae [11]. In our study, the salviaflaside content was significantly decreased by UV solar exclusion. A similar result was obtained in a previous study [21]. We speculate that β-glucosidase activities are likely involved in the metabolism of phenolic glycosides. Additionally, salviaflaside reduced the performance of *P. vulgaris* spicas, potentially because β-glucosidase activity is inhibited or depressed by photo-oxidative stress; similar results have been found in previous studies [67–69]. This result suggested that the decrease in salviaflaside upon photo-oxidative stress, which induced large amounts of H_2_O_2_, resulted in combined effects on enzymatic photodegradation and reduced enzyme production due to the decreased metabolism of the organism or decreased enzyme synthesis. This may explain the decreased salviaflaside content observed in *P. vulgaris* spicas under UV solar exclusion.

Ursolic acid and oleanolic acid are most prevalent in *P. vulgaris* spicas [4] and exhibit many bioactivities, including hepatoprotection, anti-inflammatory, antitumour, anti-hyperglycemia and antifungal effects [16, 17]. In this study, the ursolic acid and oleanolic acid contents were significantly increased under UV solar exclusion. Previous studies have shown that ursolic acid and oleanolic acid, which have similar chemical structures, demonstrate scavenging activity against ROS and inhibit peroxidation in rat liver homogenates [70, 71]. Terpenoids have been shown to possess antioxidant properties in different situations, particularly against lipid peroxidation, due to their high lipophilicity [72]. Based on this information, the enhanced ursolic acid and oleanolic acid contents of *P. vulgaris* spicas may contribute to ROS scavenging and stabilise the membrane capacity during oxidative stress under UV solar exclusion.

The antioxidant activities of ethanol extracts of *P. vulgaris* spicas were investigated by detecting ABTS·^+^and scavenging DPPH· radicals. The results suggested that all ethanol extracts from *P. vulgaris* spicas under different treatments possessed antioxidative properties. All ethanol extracts from *P. vulgaris* spicas under UV solar exclusion were more efficient at scavenging DPPH· free radicals than ethanol extracts from spicas under control treatment. This result provides evidence that ethanol extracts of *P. vulgaris* spicas subjected to photo-oxidative stress contain more antioxidants and increased the bioactive compound contents. Previous studies have shown that phenolic compounds are the major contributors to antioxidative activities [73]. The antioxidative activities of phenolic acid depends to a large extent on the compound structure, especially the number and distribution of hydroxyl groups (–OH) [74]. A compound has much higher antioxidative activity when there are two –OH groups in the ortho position (e.g., caffeic acid) [75]. Similar results were presented in earlier works [76].

## Conclusions

*Prunella vulgaris* possesses strong repair mechanisms and adapts to UV solar exclusion. Increased levels of enzymes, non-enzymatic antioxidant systems and osmolytes play crucial roles in protecting *P. vulgaris* against UV solar exclusion. There were significant increases in the phenolic and flavonoid compound contents and in the in vitro antioxidative properties of *P. vulgaris* spicas under UV solar exclusion. Our study demonstrated that *P. vulgaris* activates several antioxidant defence systems against oxidative damage caused by photo-oxidative stress.

## Methods

### Plant materials and growth conditions

This experiment was conducted on the experimental farm of the College of Pharmaceutical Sciences, Chengdu Medical College, Chengdu, located in the provincial capital of Sichuan, PR China (longitude: 104°19′E, latitude: 30°82′N, altitude: 449 m).

Seeds of *P. vulgaris* were sterilized with 9% H_2_O_2_ for 30 min followed by several rinses with distilled water, after which the seeds were planted in the experimental field of Chengdu Medical College on 25 November 2016 and subjected to regular water and fertilizer management and weeding. Then, *P. vulgaris* uniform seedlings (six-leaf stage) were transplanted into plastic pots (17-cm diameter and 12-cm height) with 850 g of nutrient soil (pH: 7.05, soil organic matter: 24.57 g kg^−1^, available-N: 85.89 g kg^−1^, available-P: 15.28 g kg^−1^, available-K_2_O: 150.27 g kg^−1^) and 3 seedlings per pot on 17 March 2017. Potted plants were placed in the experimental greenhouse with 60–70% relative humidity, 792 μmol m^−2^ s^−1^ of photosynthetically active radiation (PAR), and 22 °C/14 °C (day/night) air temperatures. The plants were inspected daily and watered as required.

On 12 May 2017, the *P. vulgaris* plants were randomly divided into two groups: the control group (CK) and UV solar exclusion group. Under ambient conditions, the light intensities of PAR and UV-B radiation were 25,000 ± 100 l× and 700 ± 20 μW cm^−2^ nm^−1^, respectively. The light intensity and UV-B radiation were measured on clear days at 10:30 and 11:00 using a TES-1335 Digital Luxmeter (Rotronic, Taipei, Taiwan) and a portable light meter (UV-340A, Lutron, Taiwan) positioned at the top of the plant canopy, respectively. UV radiation was excluded by the application of an anti-UV polyethylene film (150 μm) (Shanghai Zhuiguang Technology Co., LTD, Shanghai, China) during full-flowering stage for 13 days; this treatment allowed the transmittance of more than 92% of natural light, the exclusion of more than 80% of UV-B radiation and the exclusion of more than 99% of UV-A radiation. Under solar UV exclusion condition, the light intensity of UV-B radiation was 100 ± 10 μW cm^−2^ nm^−1^. The experiment treatments were completed on 24 May 2017.

Fresh leaves of *P. vulgaris* were collected on 24 May 2017 and then immediately frozen in liquid nitrogen and stored at − 80 °C to assess physiological and biochemical indicators. Whole plants were harvested on 5 June 2017 and dried at 70 °C for 12 h for biomass measurements. Spicas were powdered and passed through a 60-mesh sieve to determine the active ingredient contents and in vitro antioxidant capacities of ethanol extracts of *P. vulgaris*.

### Plant growth parameters

Plant growth parameters, specifically the number of branches, number of spicas, spica length and width, weight of different organs (roots, stem, leaves and spica) and whole plant weight, were measured. The spica length and width were determined with a Vernier calliper, and the weight was measured using an electronic balance (JJ124BC, Electronic Balance Test Instrument Factory, Changshu, China).

### Photosynthetic pigments and anthocyanin contents

Chlorophyll and carotenoids were extracted from 0.2 g of fresh leaf cuttings with 15 mL of 95% ethanol and then sealed and soaked in the dark at room temperature for 48 h, shaking 3–4 times; the final extract was obtained by adding 95% ethanol to a volume of 20 mL. The absorbances at 470, 649 and 665 nm, which are the wavelengths of the maximum absorbances of chlorophyll a, chlorophyll b and carotenoids in ethanol, respectively, were measured with a 752 UV–visible spectrophotometer (Shanghai Jinghua Technological Instrument, Shanghai, China). Each treatment was repeated three times. The chlorophyll a, chlorophyll b and carotenoid contents were calculated using equations and extinction coefficients according to the previous methods [23].

Anthocyanins were extracted from 0.5 g of fresh leaf cuttings with 5 mL of acidified methanol (methanol: HCl = 99:1, V:V) in the dark at room temperature for 72 h, with shaking 3–4 times. The anthocyanin content was estimated by recording the absorbances at 530 and 280 nm, respectively, using a 752 UV–visible spectrophotometer (Shanghai Jinghua Technological Instrument, Shanghai, China). The results were expressed as A g^−1^ FW. Each treatment was repeated three times.

### Peroxidase activity

Briefly, 0.5 g of fresh leaves were placed in 4.5 mL of a solution containing 0.1 mol L^−1^ phosphate buffer (pH 7.33), ground to powder in liquid nitrogen, and swirled at low temperature for full extraction. The homogenate was centrifuged at 3500 rpm for 10 min at 4 °C, and the supernatant was collected to determine the activity of POD using commercial assay kits (Nanjing Jiancheng Bioengineering Institute, Nanjing, China). Enzyme activity was determined by measuring the change in absorbance at 420 nm in units of Umg prot^−1^, defined as the amount of enzyme per microgram of tissue protein that decomposes 1 μg of enzyme catalytic substrate at 37 °C.

### Catalase and superoxide dismutase activities

Briefly, 1.0 g of fresh leaves were added to 4.0 mL of a solution containing 0.1 mol L^−1^ phosphate buffer (pH 7.33), ground to a powder in liquid nitrogen, and swirled at low temperature for full extraction. The homogenate was centrifuged at 3500 rpm for 10 min at 4 °C, and the supernatant was collected to determine the activities of CAT and SOD using commercial assay kits (Nanjing Jiancheng Bioengineering Institute, Nanjing, China). CAT activity was measured at 405 nm in Umg prot^−1^, defined as one unit of energy per milligram of tissue protein that decomposes 1 μmol of hydrogen peroxide (H_2_O_2_) per second. SOD activity was determined by measuring the change in absorbance at 550 nm in Umg prot^−1^, defined as the amount of SOD per unit of SOD activity when SOD inhibition was 50% in 1 mL of reaction solution per milligram of tissue protein.

### Ascorbate peroxidase activity

Briefly, 0.5 g of fresh leaves was added to 4.5 mL of a solution containing 0.1 mol L^−1^ phosphate buffer (pH 7.33), ground to a powder in liquid nitrogen, and swirled at low temperature for full extraction. The homogenate was centrifuged at 10,000 rpm for 10 min at 4 °C, and the supernatant was collected to determine the activity of APX using commercial assay kits (Nanjing Jiancheng Bioengineering Institute, Nanjing, China). APX activity was measured at 290 nm in units of Umg prot^−1^, defined as 1 μmol of ascorbic acid (AsA) per milligram of tissue protein per mL of reaction.

### Glutathione content

Briefly, 0.5 g of fresh leaves were added to 4.5 mL of a solution containing 0.1 mol L^−1^ phosphate buffer (pH 7.33), ground to a powder in liquid nitrogen, and swirled at low temperature for full extraction. The homogenate was centrifuged at 2500 rpm for 10 min at 4 °C, and the supernatant was collected to determine the content of GSH using commercial assay kits (Nanjing Jiancheng Bioengineering Institute, Nanjing, China). Absorbance was measured at 420 nm in milligrams of glutathione per gram of protein.

### Soluble protein, proline and malondialdehyde contents

Briefly, 0.5 g of fresh leaves were added to 4.5 mL of a solution containing 0.1 mol L^−1^ phosphate buffer (pH 7.33), ground to a powder in liquid nitrogen, and swirled at low temperature for full extraction. The homogenate was centrifuged at 3500 rpm for 10 min at 4 °C, and the supernatant was collected to determine the soluble protein, proline and MDA contents using commercial assay kits (Nanjing Jiancheng Bioengineering Institute, Nanjing, China). The soluble protein content was measured at 595 nm in g proteins l^−1^ FW. The proline content was measured at 520 nm in micrograms per gram of tissue wet weight. The MDA content was measured at 532 nm in nmol mg^−1^ proteins.

### Soluble sugar content

Briefly, 0.2 g of fresh leaves were added to 2 mL of distilled water, fully ground into a homogenate, and placed into a boiling water bath for 10 min, then cooled and centrifuged at 4000 rpm for 10 min at room temperature. The supernatant was diluted 10 times with distilled water and shaken well according to the method provided by the kit to determine the soluble sugar content (Nanjing Jiancheng Bioengineering Institute, Nanjing, China). The soluble sugar content was measured at 620 nm in mg mg^−1^ proteins.

### Hydrogen peroxide content

Briefly, 0.5 g of fresh leaves were added to 4.5 mL of a solution containing 0.1 mol L^−1^ phosphate buffer (pH 7.33), ground to a powder in liquid nitrogen, and swirled at low temperature for full extraction. The homogenate was centrifuged at 10,000 rpm for 10 min at 4 °C, and the supernatant was collected to determine the H_2_O_2_ content using commercial assay kits (Nanjing Jiancheng Bioengineering Institute, Nanjing, China). The H_2_O_2_ content was measured at 405 nm in millimoles per milligram of protein.

### Total phenolic content

One gram of dried spica powder, weighed precisely, was combined with 10 mL of 80% ethanol, and reflux extraction was performed for 1.5 h at 90 °C and repeated three times. Reaction conditions were optimized at ambient temperature. One millilitre of the above-mentioned extract solution was mixed with 0.5 mL of Folin–Ciocalteu reagent and 1.7 mL of 20% Na_2_CO_3_ in a test tube for 60 min. The absorbance was measured at 760 nm using a 752 UV–visible spectrophotometer (Shanghai Jinghua Technological Instrument, Shanghai, China). Gallic acid was used to generate a standard calibration curve, and the results were expressed as milligrams of gallic acid equivalents per 100 mg of spica dry weight (%).

### Total flavonoid content

One gram of dried spica powder was extracted three times with 10 mL of 35% ethanol solution in a water bath for 3.5 h at 86 °C. The total flavonoid content in *P. vulgaris* was determined by a spectrophotometric method using a 752 UV–visible spectrophotometer (Shanghai Jinghua Technological Instrument, Shanghai, China) measuring the absorbance at 510 nm, using rutin as the standard. Reaction conditions were optimized at ambient temperature and in the dark. One millilitre of the above-mentioned extract solution was combined with 0.7 mL of 50 mg mL^−1^ NaNO_3_ and reacted for 7 min. Next, 0.3 mL of 100 mg mL^−1^ AlCl_3_ was mixed with the solution for 6 min, and 5.0 mL of 1 mol L^−1^ NaOH solution was added. The results were expressed as milligrams of rutin equivalents per 100 mg of spica dry weight (%).

### Total polysaccharide content

*Prunella vulgaris* dried spica power (0.2 g) was refluxed three times with 100 mL of 95% ethanol for 3 h at 95 °C, and the residue was thoroughly washed with 95% ethanol to remove ethanol-soluble constituents. Then, the residue was refluxed with an appropriate amount of petroleum ether at 80 °C for 0.5 h to de-colour and achieve petroleum ether recovery. Next, the residue was sequentially extracted in a hot water bath with 50 mL, 20 mL, and 20 mL of distilled water for 0.5 h at 40 °C. The polysaccharide content was determined using a 752 UV–visible spectrophotometer (Shanghai Jinghua Technological Instrument, Shanghai, China) measuring the absorbance at 490 nm with glucose as the standard. One millilitre of the above-mentioned extract solution was mixed with 1 mL of distilled water and 1 mL of 6% phenol solution; then, 5 mL of concentrated sulfuric acid was rapidly added, shaken and reacted at ambient temperature for 5 min, in a boiling water bath for 15 min, and then cooled to room temperature to determine the content. The results were expressed as milligrams of rutin equivalents per 100 mg of spica dry weight (%).

### Rosmarinic acid, salviaflaside, caffeic acid and hyperoside contents

Two grams of dried spica powder was mixed with 15 mL of 80% methanol in an ultrasonic bath for 35 min at ambient temperature, and then the extracted solution was centrifuged at 12,000 rpm for 15 min. The supernatant was passed through a 0.45-μm organic membrane filter prior to HPLC analysis. A Dionex UltiMate 3000 HPLC System (Dionex Corp., Sunnyvale, CA, USA) equipped with a diode array detector (DAD-3000) and a Uranus C18 column (250 mm × 4.6 mm) was used to quantify the extracts (10 μL). The mobile phase consisted of chromatographic methanol (solvent A) and 0.2% NaH_2_PO_4_ solution (solvent B), the flow rate was 0.8 mL min^−1^, and the following multilinear gradient was applied: 20–40% A (0–20 min), 40–70% A (20–35 min), 70–90% A (35–45 min), and 90–20% A (45–60 min). The column oven temperature was set to 30 °C with a run time of 60 min. UV wavelengths were monitored at 325 nm for caffeic acid salviaflaside and rosmarinic acid and 360 nm for hyperoside. The identification of each peak was based on the retention time and the chromatography of the authentic standards. Concentrations of phenolic acids and flavonoid compounds were calculated according to the calibration curves of the standards, and the results were presented as milligrams per 100 mg of spica dry weight (%).

### Ursolic acid and oleanolic acid contents

One gram of dried spica powder was combined with an appropriate amount of ether, soaked overnight, and refluxed for 3 h at 45 °C until the residue was colourless. The residue was then soaked with petroleum ether two times, the residual solvent was evaporated, and the extracts were dissolved with methanol. The extracted solution was passed through a 0.45-μm organic membrane filter prior to HPLC analysis. A Dionex UltiMate 3000 HPLC System (Dionex Corp., Sunnyvale, CA, USA) equipped with a diode array detector (DAD-3000) and a Uranus C18 column (250 mm × 4.6 mm) was used to quantify the extracts (10 μL). The mobile phase consisted of chromatographic methanol (90%) and 5% CH_3_COONH_4_ solution (10%), the flow rate was 0.6 mL min^−1^, the column oven temperature was 25 °C, and the detection wavelength was 210 nm, with a run time of 35 min. The ursolic acid and oleanolic acid contents were calculated according to the calibration curves of the standards, and the results were presented as milligrams per 100 mg of spica dry weight (%).

### DPPH· free radical scavenging assay

One gram of dried spica powder was extracted by ultrasound with 30 mL of 70% ethanol solution for 30 min at 70 °C. One millilitre of the above-mentioned extract solution was mixed with 4.0 mL of 0.004% DPPH· solution and maintained at room temperature in the dark for 30 min. The absorbance was measured at 517 nm using a 752 UV–visible spectrophotometer (Shanghai Jinghua Technological Instrument, Shanghai, China) against a methanol blank. The results were expressed as the percentage of DPPH· radical inhibition (%). All tests and analyses were run three times and averaged. Calculations were based on the following equation:

$${\text{DPPH}} \cdot \;{\text{scavenging}}\;{\text{effect}}\left( \% \right) = \left[ {{\text{A}}0 - \left( {{\text{A}}1 - {\text{A}}2/{\text{A}}0} \right)} \right] \times 100\%$$

where A0 is the absorbance of the DPPH· solution without sample (4 mL of DPPH· + 1 mL of methanol), A1 is the absorbance of the test sample mixed with DPPH· solution (4 mL of DPPH· + 1 mL of sample) and A2 is the absorbance of the sample without DPPH· solution (4 mL of methanol + 1 mL of sample).

### Trolox equivalent antioxidant capacity (TEAC assay)

One gram of dried spica powder was extracted by ultrasound with 30 mL of 70% ethanol solution for 30 min at 70 °C. A 0.1-mL aliquot of the above-mentioned extract solution was mixed with 3.9 mL of working fluid (7 mmol L^−1^ ABTS·^+^) and with 2.45 mmol L^−1^ K_2_S_2_O_8_, incubated in the dark at 23 °C for 12–16 h, then diluted in buffer (pH 7.4) until its absorbance was 0.700 ± 0.005 at 734 nm. The solution was then incubated at 23 °C for 6 min, and the absorbance was read at 734 nm using a 752 UV–visible spectrophotometer (Shanghai Jinghua Technological Instrument, Shanghai, China). The percent inhibition was calculated against a blank control. All tests and analyses were run three times and averaged. Calculations were based on the following equation:

$${\text{ABTS}}\cdot^{ + } \;{\text{scavenging}}\;{\text{effect}}\left( \% \right) = \left[ {\left( { 1{-}{\text{A1}}} \right)/{\text{A2}}} \right] \times 100\%$$

where A1 is the absorbance of the test sample mixed with working solution (3.9 mL of working solution + 0.1 mL of sample) and A2 is the absorbance of working solution without sample (3.9 mL of working solution + 0.1 mL of water).

Trolox was used to generate a standard calibration curve, and the results were expressed as TEAC. The concentration of antioxidants giving the same percent inhibition of ABTS·^+^ as that of 1 mM Trolox was considered TEAC.

### Statistical analyses

Data are presented as the mean ± SD. Significant differences were determined by one-way analysis of variance (ANOVA) followed by Duncan’s multiple range test using SPSS 17.0 software (SPSS, Chicago, IL, USA).

## Abbreviations

APX: ascorbate peroxidase
AsA: ascorbic acid
CAT: catalase
DAD: diode array detector
DPPH: 1, 1-diphenyl-2-picrylhydrazyl
GSH: glutathione
H_2_O_2_: hydrogen peroxide
MDA: malondialdehyde
POD: peroxidase
ROS: reactive oxygen species
SOD: superoxide dismutase
TEAC: trolox equivalent antioxidant capacity
UV: ultraviolet
